# Profil anthropometrique des enfants scolarises tananariviens

**DOI:** 10.11604/pamj.2013.16.62.3087

**Published:** 2013-10-22

**Authors:** Fetralinjiva Razafimanantsoa, Notahiana Razafindramaro, Hasina Raherimandimby, Annick Robinson, Olivat RakotoAlson, Andry Rasamindrakotroka

**Affiliations:** 1 Service de Biologie, CHU Befelatanana, Antananarivo, Madagascar; 2 Service de Médecine interne, CHU Befelatanana, Antananarivo, Madagascar; 3 Service de Biologie, CHR Fianarantsoa, Madagascar; 4 Service de Pédiatrie, CHME Tsaralalana, Antananarivo, Madagascar; 5 Service de Biologie, CHU RavoahangyAndrianavalona, Antananarivo, Madagascar

**Keywords:** Enfant, taille, indice de mass corporelle, z-score, Madagascar, children, height, body mass index, z-score, Madagascar

## Abstract

Les enfants tananariviens sont en état de malnutrition chronique. Notre objectif est d’évaluer l'indice de masse corporelle (IMC) pour estimer les enfants apparemment "sains". Une enquête et une mesure de la taille et du poids des enfants scolarisés tananariviens de 6 à 11 ans ont été réalisées. Après leur accord, la taille et l'indice de masse corporelle des 442 enfants tirés au hasard ont été ainsi obtenus. L'analyse de la moyenne de la taille a révélé une différence significative à 8 ans, une différence non évidente sur l'indice de masse corporelle. La comparaison avec les valeurs de référence (OMS 2006) a montré un retard statural de 34% avec une tendance globale à la hausse et un déficit pondéral égal à 5,5% selon le z score. Ainsi, au sein d'une population malnutrie, l'indice de masse corporelle pourrait être utilisé comme un des paramètres à considérer dans l’évaluation de l’état de santé pour classer ces enfants en bonne santé apparente.

## Introduction

La malnutrition constitue un problème de santé publique dans le monde et en particulier dans les pays en voie de développement [1]. Elle peut revêtir, entre autre, la forme d'une émaciation, d'un retard de croissance, d'une insuffisance ou d'un excès pondéral. L'indicateur anthropométrique taille-pour-âge permet d’évaluer la malnutrition chronique ou le retard de croissance en utilisant le z score ou le percentile par rapport à la médiane de la population de référence.

A Antananarivo, capitale de Madagascar, l'enquête démographique sanitaire (nutritionnelle nationale) en 2009 a révélé que 46,8% des enfants de moins de cinq ans souffrent d'une malnutrition chronique. La prévalence de cet état augmente avec l’âge [2]. On considère qu'après l’âge de 2 ans, le retard de croissance staturale acquis dès les plus jeunes âges est difficilement rattrapable. Il est alors primordial d'avoir des indicateurs d'identification d'individu « sain » dans cet état de chronicité. L'indice poids-pour-taille est difficile à interpréter pendant l'adolescence. L'indice poids-pour-âge peut être affecté par la situation nutritionnelle actuelle. L'indice de masse corporelle (IMC), basé sur des mesures simples et standardisées et prenant en compte trois variables pourrait être une méthode plus précise même s'il n'a été recommandé que pour évaluer le degré d'obésité ou le déficit énergétique chronique.

Aussi, la présente étude s'est-elle fixée comme objectif d’évaluer la place de l'indice de masse corporelle pour estimer les enfants scolarisés tananariviens en bonne santé apparente.

## Méthodes

Une étude prospective, transversale a été réalisée en 2009 dans les établissements scolaires primaires de la Commune Urbaine d'Antananarivo. Elle a concerné les élèves en bonne santé apparente de 6 ans, âge officiel d'entrée en école primaire, à 11 ans, âge de passer au collège. Au sein des établissements privés et publics de la commune urbaine d'Antananarivo, un échantillonnage systématique a été effectué sur la liste des établissements fournis par la circonscription scolaire de ladite commune. Un intervalle d’échantillonnage différent a été adopté, 20 pour les établissements publics et 40 pour les établissements privés en raison de leur nombre cinq fois plus élevé. Au sein de chaque école sélectionnée, un deuxième échantillonnage aléatoire simple sans remplacement a été réalisé en fonction du genre pour ne prendre que les 1/4 des élèves.

Après les informations fournies préalablement aux parents et l'obtention de leur consentement, un remplissage de questionnaire et une mesure du poids et de la taille ont été effectués pour chaque élève retenu. Ont été exclus les enfants présentant un ‘dème, ou une rate ou un foie palpable et ceux ayant une température supérieure à 38,5°C au moment de l'enquête. Le registre scolaire a fourni le sexe et la date de naissance des élèves. Le poids et la taille chez les enfants légèrement vêtus ont été mesurés respectivement avec une balance pèse-personne et un statiomètre fixe. L'indice de masse corporelle (IMC) a été calculé selon la formule IMC= P (kg)/ T2 (m2). La moyenne et l’écart-type de l'IMC et de la taille en fonction de l’âge et du sexe ont été calculés. La valeur du z score de chaque individu pour la taille sur âge et pour l'IMC sur âge a été évaluée en prenant les valeurs de référence fournies par l'OMS 2006 [3]. Tout enfant ayant un z score < -2 a été considéré comme présentant un retard de croissance staturale et un déficit pondéral respectivement. Le test de student pour la moyenne et le test de Chi2 pour les pourcentages avec un seuil de signification à 0,05 ont été utilisés pour évaluer la différence. La saisie et le calcul des données ont été faits sur Excel 2003.

## Résultats

**Figure 1:**
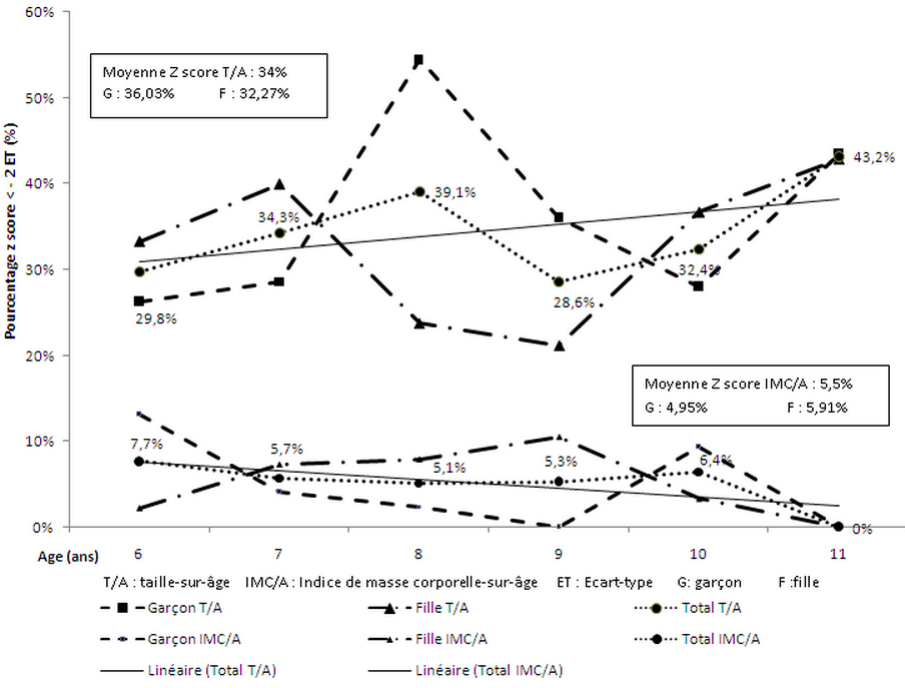
Prévalence du retard de croissance et du déficit pondéral

Au total, 442 élèves issus de 6 établissements publics et de 10 établissements privés de niveau primaire sont colligés dans cette étude. L’âge moyen en mois des garçons et des filles était respectivement de 104 mois (+/-19,5) et de 101 mois (+/-18,1). Aucune différence n'a été constatée entre les deux établissements que ce soit sur les données anthropométriques générales que sur le z-score. La statistique descriptive générale de la taille et de l'IMC des élèves a révélé un retard de croissance staturale et un déficit pondéral chez les individus de genre masculin. La différence de taille à 8 ans a été significative (p< 0,01) en faveur des individus de sexe féminin. Néanmoins, cette différence n’était pas évidente pour l'IMC (Tableau 1:). Par ailleurs, l’évaluation du retard de croissance avec la courbe du z score a montré que les individus de sexe masculin (36%) étaient plus affectés que ceux du sexe opposé (32%) bien que la différence n’était pas significative (p > 0,05) et quelque soit l’établissement. Par contre, la prévalence du déficit pondéral masculin était plus faible (5% vs 6%) (Figure 1).

**Tableau 1:** Taille et indice de masse corporelle des élèves tananariviens de 6 à 11 ans

			Taille (cm)		IMC (kg/m^2^)	
	Effectif		Moyenne ( Ecart-type)		Moyenne ( Ecart-type)	
Age(année)	Masculin	Féminin	Masculin	Féminin	Masculin	Féminin
6	38	45	111,6(4,33)	111,1(5,24)	14,7(0,94)	14,6(0,94)
7	49	55	116,7(4,72)	115,5(5,39)	14,8(0,97)	14,6(1,17)
8	44	38	118,5(7,5)**	122,5(5,99)**	15,2(1,29)	15,4(1,94)
9	36	38	125,8(5,27)	126,4(5,45)	15,3(0,91)	15,1(1,6)
10	32	30	131(6,07)	131,1(7,74)	15,5(1,13)	15,9(1,45)
11	23	14	132,7(6,34)	136,4(7,76)	15,9(0,92)	16,1(1,29)
**Total**	222	220	122,6(5,07)	123,8(6,26)	15,2(1,02)	15,3(1,39)

** p< 0,05

## Discussion

L'aspect transversal, le nombre d’échantillon analysé pour certaines catégories d’âge ainsi que la limite géographique de la population concernée constituent les limites de cette étude. Mais, d'une façon générale, le z score des élèves urbains tananariviens a montré que 34% ont eu un retard de croissance tandis que seul 5,5% a présenté un déficit pondéral.

En analysant la taille des ces élèves, nous avons constaté que la taille des garçons était plus petite. Un développement statural féminin important a été noté à 8 ans et à 11 ans. Cette poussée staturale féminine survenue avant celle des garçons est connue [4]. La taille des enfants tananariviens était semblable aux enfants indiens [5] mais faible par rapport aux ruraux sud-africains [6] et aux mozambicains [7]. La raison serait probablement d'ordre génétique.

La comparaison de la taille avec les valeurs de référence de l'OMS a montré d'une façon générale que 34% des enfants tananariviens avaient un retard de croissance selon le z score. Ce taux était légèrement supérieur par rapport aux enfants marocains [8] mais faible par rapport aux enfants tanzaniens [9]. Nous avons noté également une augmentation de la tendance globale du déficit statural au fil des années.

En analysant le deuxième indicateur, l'IMC, les moyennes entre filles et garçons n’étaient pas différentes. L'analyse combinée de la taille et de l'IMC a permis d'avancer qu’à 8 ans et 11 ans, l'augmentation staturale importante était proportionnelle au gain pondéral. Ainsi, quelque soit la taille des enfants, l'IMC peut révéler une autre forme d’état sanitaire. L'organisme s'adapte à son état de malnutrition chronique, s'il en a, en agissant en même temps et sur la taille et sur le poids. L'IMC moyen des enfants urbains scolarisés tananariviens tout sexe confondu était plus grand que l'IMC moyen des enfants ruraux sud-africains [6] et celui des enfants indiens de sexe masculin [5]. Par contre, l'IMC des enfants mozambicains a dépassé le notre à 10 ans [7].

La comparaison de l'IMC avec les valeurs de référence de l'OMS a montré d'une façon générale que seuls 5,5% des enfants tananariviens ont eu un déficit pondéral selon le z score. En plus la tendance globale du déficit pondéral a diminué au cours de l’âge tendant vers une différence nulle. Cette prévalence de déficit pondéral a été légèrement inférieure par rapport aux enfants marocains de 8,6% [8]. Toutefois ce seuil est au-dessus de 2,8%, part accordée par l'OMS aux enfants à z score< -2. Aussi, notre état de malnutrition ou d'adaptation n’était pas totalement acceptable.

## Conclusion

Les enfants scolarisés tananariviens ont présenté de façon générale un retard de croissance de l'ordre de 34% dû à une malnutrition chronique. Par contre l'indice de masse corporelle sur âge en comparaison avec les valeurs de référence de l'OMS 2006 selon le z score n'a montré qu'un déficit pondéral de l'ordre de 5%. Ainsi, il paraît que l'IMC peut être utilisé parmi les facteurs qui peuvent être retenus dans l'appréciation de son état de bonne santé si son z score par rapport à la valeur fournie par l'OMS est normale. En d'autres termes, malgré le problème nutritionnel, l'IMC serait un meilleur indicateur de l’état nutritionnel voire de l’état sanitaire pour les enfants tananariviens.
